# Cross-Modulation of Homeostatic Responses to Temperature, Oxygen and Carbon Dioxide in *C. elegans*

**DOI:** 10.1371/journal.pgen.1004011

**Published:** 2013-12-19

**Authors:** Eiji Kodama-Namba, Lorenz A. Fenk, Andrew J. Bretscher, Einav Gross, K. Emanuel Busch, Mario de Bono

**Affiliations:** MRC Laboratory of Molecular Biology, Cambridge, United Kingdom; University of California San Diego, United States of America

## Abstract

Different interoceptive systems must be integrated to ensure that multiple homeostatic insults evoke appropriate behavioral and physiological responses. Little is known about how this is achieved. Using *C. elegans*, we dissect cross-modulation between systems that monitor temperature, O_2_ and CO_2_. CO_2_ is less aversive to animals acclimated to 15°C than those grown at 22°C. This difference requires the AFD neurons, which respond to both temperature and CO_2_ changes. CO_2_ evokes distinct AFD Ca^2+^ responses in animals acclimated at 15°C or 22°C. Mutants defective in synaptic transmission can reprogram AFD CO_2_ responses according to temperature experience, suggesting reprogramming occurs cell autonomously. AFD is exquisitely sensitive to CO_2_. Surprisingly, gradients of 0.01% CO_2_/second evoke very different Ca^2+^ responses from gradients of 0.04% CO_2_/second. Ambient O_2_ provides further contextual modulation of CO_2_ avoidance. At 21% O_2_ tonic signalling from the O_2_-sensing neuron URX inhibits CO_2_ avoidance. This inhibition can be graded according to O_2_ levels. In a natural wild isolate, a switch from 21% to 19% O_2_ is sufficient to convert CO_2_ from a neutral to an aversive cue. This sharp tuning is conferred partly by the neuroglobin GLB-5. The modulatory effects of O_2_ on CO_2_ avoidance involve the RIA interneurons, which are post-synaptic to URX and exhibit CO_2_-evoked Ca^2+^ responses. Ambient O_2_ and acclimation temperature act combinatorially to modulate CO_2_ responsiveness. Our work highlights the integrated architecture of homeostatic responses in *C. elegans*.

## Author Summary

Many animals are either attracted or repelled by carbon dioxide. We show that the way *C. elegans* responds to CO_2_ depends on the temperature it has acclimated to and the oxygen tensions it is experiencing. The effects of acclimation temperature are mediated by a temperature-sensing neuron called AFD that also responds to CO_2_. The responses evoked in AFD by a change in CO_2_ concentration are reprogrammed by acclimation temperature. This reprogramming does not appear to require synaptic input from other neurons. O_2_ modulates CO_2_ avoidance by setting the activity of the tonically signalling O_2_ sensor URX. A switch from 21% to 19% O_2_ is sufficient to convert CO_2_ from a neutral stimulus to an aversive one in a *C. elegans* wild strain. Modulation of CO_2_ responses by O_2_ cues requires the interneuron RIA which itself responds to changes in CO_2_ and is directly post-synaptic to URX. CO_2_ gradients are likely to be common in rotting fruit where *Caenorhabditis* live. Such gradients could be associated with food, pathogens, conspecifics or predators of *C. elegans*. The value of CO_2_ as a sensory cue thus depends crucially on context. This may explain the remarkable complexity of CO_2_ sensing in *C. elegans*.

## Introduction

To maintain a constant internal milieu animals use internal sensory receptors to monitor cues such as CO_2_/pH [1], O_2_ [2], temperature [3], and osmolality [4]. These interoceptors counter changes in internal milieu by coordinating homeostatic responses that alter physiology and behavior [5]. Cross-talk between different interoceptive systems is likely to be important to ensure an integrated homeostatic response by the animal to multiple homeostatic insults. However, relatively little is known, at the molecular and circuitry levels, about how such cross-talk is encoded.

In vertebrates electrophysiological studies have identified cell populations and circuits that respond to homeostatic imbalance in O_2_, CO_2_/pH and temperature. The neurons comprising these circuits are only beginning to be resolved, and the molecular mechanisms controlling their responses are poorly understood. Nevertheless, studies in several animals suggest that cross-modulation of homeostatic responses is important for survival. In panting mammals, a rise in core body temperature elicits increased ventilation rate to help cooling, even though this causes temporary alkalosis of the blood due to excessive blowing off of CO_2_. This over-ride appears to be achieved by changing the set-point at which CO_2_ sensors inhibit ventilation when [CO_2_] decreases, but the mechanisms involved are unclear [6]. In the mouse, recent work has shown that suppressing the activity of serotonergic neurons impairs both respiratory and body temperature control, although whether the same or different sub-populations of neurons mediate these effects is unclear [7], [8]. In mammals, the drive to increase ventilation rate is stimulated more strongly when animals simultaneously experience a drop in O_2_ and a rise in CO_2_ [9].

In invertebrates, such as the free-living nematode *C. elegans*, behavioral mechanisms that counter homeostatic imbalance are particularly important, since the animal's buffering capacity is limited. *C. elegans* responds to variation in temperature, O_2_ and CO_2_ by mounting sophisticated behavioral responses. Exposure to temperatures above or below the range in which *C. elegans* can grow elicits strong avoidance responses [10]. When navigating thermal clines in which it can thrive, ∼15°C to 25°C, *C. elegans* migrates to the temperature at which it grew recently, as long as this was not associated with starvation [11], [12]. These responses require the animal to memorize its recent temperature experience and to change this memory when temperature or nutrient conditions change. A neural circuit that subserves these behaviors has been identified, and involves the thermosensory neurons AFD and AWC [13]–[16]. Temperature experience alters the thermosensing properties of AFD neurons: in animals acclimated to higher temperatures, the threshold at which a temperature rise evokes a Ca^2+^ response in AFD occurs at correspondingly higher temperatures [17], [18]. This plasticity allows animals to respond homeostatically to external temperature fluctuations, by seeking and remaining at temperatures they are acclimated to.

*C. elegans* also displays responses to variation in [O_2_], and avoids both high and low O_2_ [19]. Wild-caught *C. elegans* strongly avoids 21% O_2_, both on and off food, and burrow to escape from the surface [20]. This avoidance response is sculpted by O_2_-sensing neurons in the body cavity called AQR, PQR and URX [20], [21], [22]. When [O_2_] levels rise towards 21% the AQR, PQR and URX neurons become activated, by a mechanism involving the atypical soluble guanylate cyclases GCY-35/GCY-36. The tuning of the O_2_ response is sharpened by a neuroglobin expressed in AQR, PQR and URX neurons, called GLB-5, that suppresses neuronal activity when ambient [O_2_] falls just below 21% [20], [23]. The AQR, PQR and URX neurons are all tonic receptors: they show sustained signalling as long as [O_2_] is high [24]. This tonic activity stimulates sustained rapid movement until animals encounter a preferred lower [O_2_] environment.

*C. elegans* also avoids elevated CO_2_ [25], [26]. As in vertebrates, high [CO_2_] is harmful to *C. elegans*, reducing brood size and disrupting muscle structure [27]. An array of sensory neurons mediates CO_2_ avoidance behavior [28]. This network includes the temperature sensor AFD, the major gustatory neuron ASE, and the BAG neurons, which are also activated by decreasing O_2_ levels [22].

Here we investigate how the temperature and O_2_ sensing systems of *C. elegans* modulate the distributed circuit that mediates responses to CO_2_.

## Results

### Previous temperature experience sets CO_2_ avoidance in *C. elegans*

**Figure 1:**
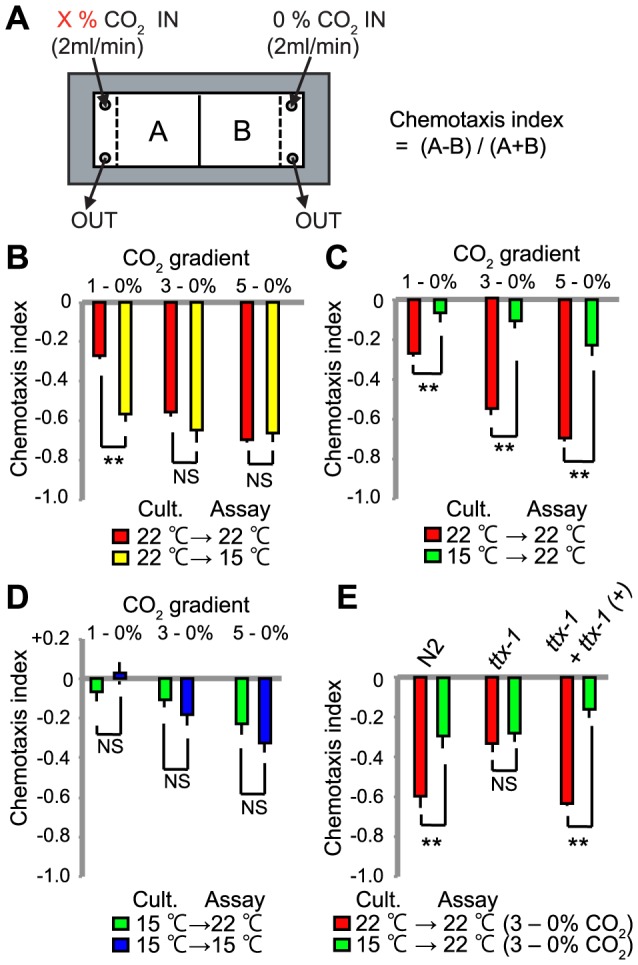
CO_2_ avoidance is modulated by acclimation temperature. A. Assay for *C. elegans* CO_2_ responses. Animals navigate a defined CO_2_ gradient in a microfluidic device. The chemotaxis index is calculated by counting animals in two halves of the device, using the formula shown. B–D. Chemotaxis indices for animals cultivated at either 15°C or 22°C and assayed in different CO_2_ gradients at either 15°C or 22°C. **, *p*<0.01; n.s., not significant, Student's *t*-test. E. A mutation in *ttx-1*, which is specifically required to confer AFD neural identity, disrupts modulation of CO_2_ avoidance by acclimation temperature. Assays were performed in 3%–0% CO_2_ gradients. **, *p*<0.01; n.s., not significant, Student's *t*-test.

To examine if temperature can modify *C. elegans'* responses to CO_2_ we grew N2(Bristol) animals at 22°C and compared their behavior in CO_2_ gradients at 15°C and 22°C (Figure 1A, B) [25], [28]. CO_2_ avoidance at the two temperatures was similar when animals navigated 3%–0% and 5%–0% CO_2_ gradients. However, animals in a 1–0% CO_2_ gradient avoided the high CO_2_ half of the microfluidic device more strongly when assayed at 15°C compared to 22°C (Figure 1A).

*C. elegans* can retune its temperature preference according to the temperature to which it is acclimated [13], [29]. This behavior is encoded in AFD [17], [18], a neuron that also responds to CO_2_ [28]. We therefore examined how previous temperature experience altered subsequent CO_2_ responses. We grew animals at 15°C or 22°C, and assayed their CO_2_ responses at each temperature. Strikingly, previous temperature experience altered CO_2_ avoidance. Animals grown at 15°C avoided CO_2_ less strongly than animals grown at 22°C, regardless of whether the assay temperature was 15°C or 22°C (Figure 1B–D). Animals grown at 15°C showed weaker CO_2_ avoidance even when exposed to relatively high CO_2_ levels, 5% (Figure 1B–D). Thus, the temperature to which *C. elegans* has acclimated helps determine the aversiveness of CO_2_.

### Acclimation temperature does not reprogram CO_2_ responses in AFD-defective mutants

We investigated if the AFD neurons helped to reprogram CO_2_ avoidance behavior according to acclimation temperature. The *ttx-1* (*t*hermo*t*a*x*is defective) gene encodes a member of the OTD/OTX subclass of homeodomain transcription factors [30]. Mutations in *ttx-1* selectively disrupt AFD specification, and confer a thermotaxis-defective phenotype. Loss of *ttx-1* also reduces CO_2_ avoidance in animals navigating CO_2_ spatial gradients [28]. If AFD neurons were important for temperature regulation of CO_2_ avoidance responses, then *ttx-1* mutants would display similar CO_2_ avoidance regardless of cultivation temperature. As shown previously, *ttx-1* mutants grown at 22°C only avoided CO_2_ weakly [28], resembling wild-type animals grown at 15°C (Figure 1E). This defect was rescued by a wild-type *ttx-1* transgene (Figure 1E). By contrast, loss of *ttx-1* did not alter the CO_2_-avoidance behavior of animals cultivated at 15°C. These data suggest AFD is required for acclimation temperature to modify CO_2_ aversive responses.

### Acclimation temperature re-programs the CO_2_ responsiveness of AFD

**Figure 2:**
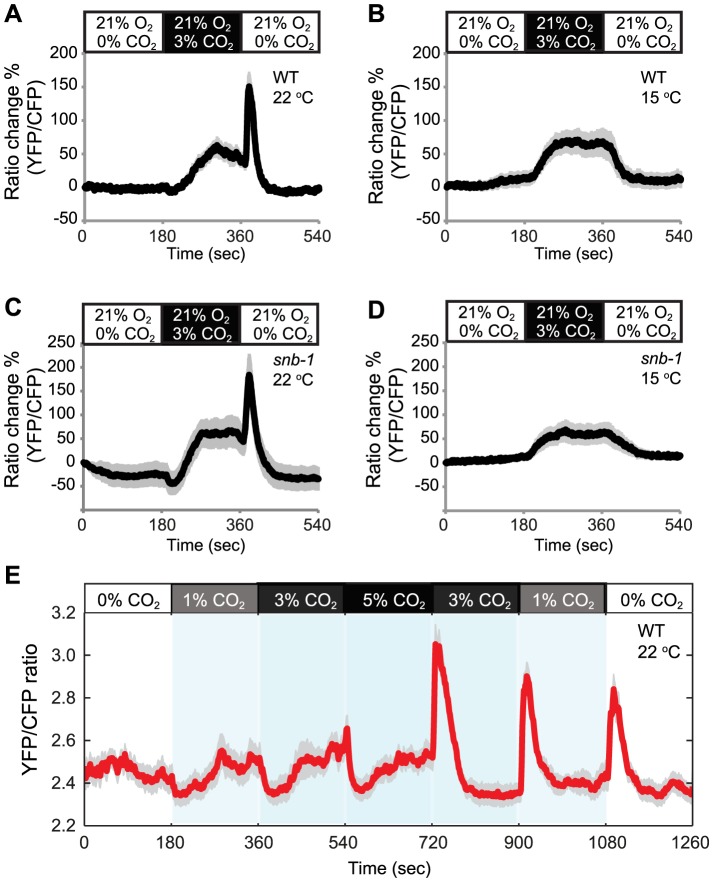
Acclimation temperature alters CO_2_-evoked Ca^2+^ responses in AFD neurons. In animals cultivated at 22°C a rise and fall in CO_2_ evokes a complex Ca^2+^ response in AFD neurons (A). Ca^2+^ initially falls when CO_2_ begins to increase, then rises. When CO_2_ levels fall, there is a Ca^2+^ spike. By contrast, animals cultivated at 15°C show a simple response to the same stimulus (B). C–D The effect of acclimation temperature on CO_2_-evoked Ca^2+^ responses in AFD neurons is unaltered in *snb-1* mutants defective in synaptobrevin. E. Ca^2+^ responses evoked in AFD by a 0%–1%–3%–5%–3%–1%–0% CO_2_ stimulus train in animals acclimated to 22°C. Shading highlights switch times. Acclimation temperature is shown for each panel under genotype.

Acclimation temperature sets the response threshold of AFD neurons to warming [17]. This prompted us to investigate whether acclimation temperature also alters the CO_2_ responsiveness of AFD. To measure CO_2_-evoked Ca^2+^ responses in AFD we expressed the genetically encoded Ca^2+^ sensor cameleon YC3.60 [31] from the *gcy-8* promoter [32]. For our recordings we used animals acclimated to 15°C or 22°C, but maintained animals at 22°C while we imaged them. In animals acclimated to 22°C high CO_2_ evoked in AFD the complex Ca^2+^ response described previously (Figure 2A) [28]. This typically consisted of an initial slight drop in Ca^2+^ when CO_2_ levels rose, followed by a rise in Ca^2+^ to above pre-stimulus levels, and finally, when the CO_2_ stimulus was removed, a Ca^2+^ spike that rapidly decayed back to baseline. By contrast, animals acclimated to 15°C exhibited a simple response: a rise in Ca^2+^ when CO_2_ levels rose, and a fall when CO_2_ was removed (Figure 2B). These data suggest that the previous temperature experience of *C. elegans* reconfigures the CO_2_ response properties of AFD neurons.

To investigate if this retuning was driven by the intrinsic temperature-sensing properties of AFD neurons, or required pre-synaptic input, we imaged the Ca^2+^ responses of AFD neurons to CO_2_ in *snb-1* (*s*y*n*apto*b*revin*-1*) mutants, which are defective in synaptic transmission [33]. CO_2_–evoked responses in AFD neurons were not altered in *snb-1* animals compared to wild type, regardless of acclimation temperature (Figure 2C, D). These data suggest that the temperature experience can retune the CO_2_ response properties of AFD neurons when synaptic signalling is defective.

We characterized the response properties of the AFD neurons further. Previously, we had only exposed animals to sharp changes in CO_2_ that occurred within 1–2 s, and we always returned animals to 0% CO_2_ between stimuli [29]. To examine AFD responses to rises in CO_2_ from non-zero levels, we subjected animals acclimated to 22°C to a stimulus train involving multiple CO_2_ switches, namely 0%–1%–3%–5%–3%–1%–0%. Whenever CO_2_ levels increased, we observed an initial drop in Ca^2+^ followed by a rise in Ca^2+^ (Figure 2E). Whenever CO_2_ levels decreased, we observed a spike of Ca^2+^ that rapidly returned to baseline. This pattern of CO_2_ evoked Ca^2+^ response suggests that AFD can encode whether an animal is moving towards higher or lower CO_2_.

Previous work has identified one potential molecular sensor for CO_2_, the transmembrane guanylate cyclase *gcy-9* [34]. We compared CO_2_-evoked responses in AFD neurons in wild type and *gcy-9* mutants. We observed no difference in the response, suggesting that molecules other than GCY-9 confer CO_2_-responsiveness to AFD neurons (Figure S1).

### AFD responses to CO_2_ are reconfigured by the steepness of the CO_2_ gradient

**Figure 3:**
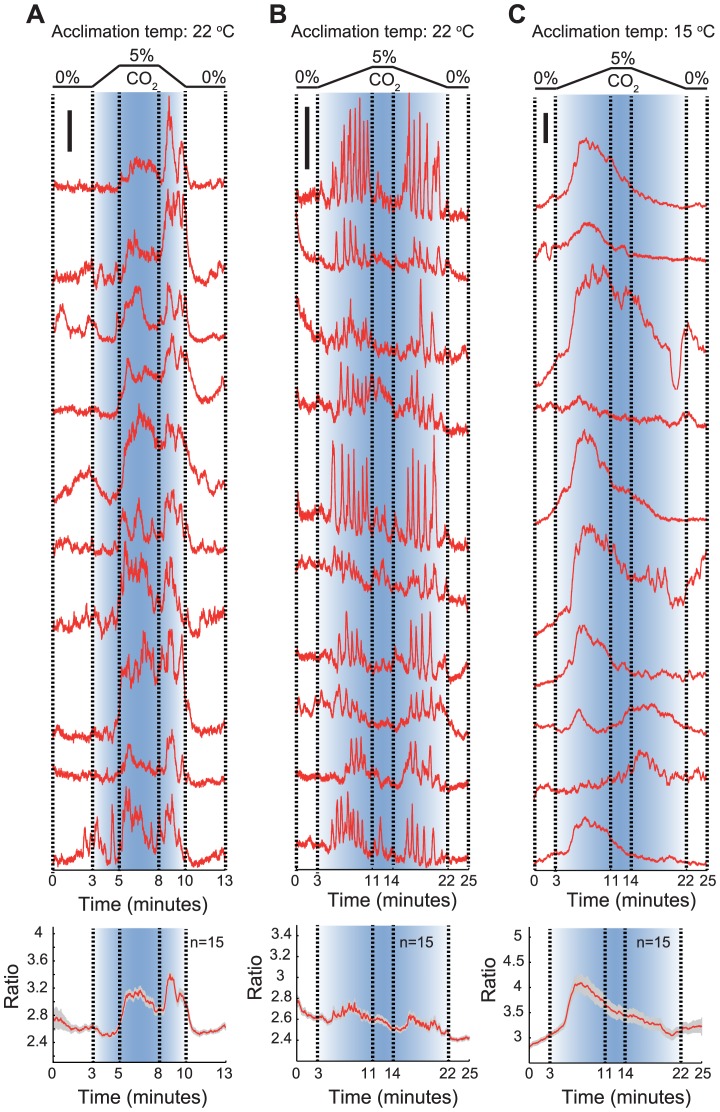
Shallow and steep CO_2_ gradients evoke qualitatively different Ca^2+^ responses in AFD. A. Ca^2+^ responses evoked in AFD by CO_2_ switches indicated at top, involving linear 0–5% and 5%–0% CO_2_ gradients occurring over 2 minutes. This corresponds to a rate of change of 0.04% CO_2_/second. The upper part of the panel shows traces obtained from 10 randomly selected individual AFD neurons; an average trace is plotted at the bottom. Animals imaged in this panel were acclimated to 22°C. B, C. Ca^2+^ responses evoked in AFD by CO_2_ switches indicated at top, involving linear switches from 0–5% and 5%–0% CO_2_ occurring over 8 minutes. This corresponds to a change of 0.01% CO_2_/second. The upper part of the panels shows traces obtained from 10 randomly selected individual AFD neurons; average traces are plotted at the bottom. Animals imaged in (B) were acclimated to 22°C; those in (C) were acclimated at 15°C. For each panel, individual and average traces are at the same scale. The scale bar in each panel represents 0.4 YFP/CFP ratio unit.

The ubiquity of CO_2_ suggests that its value as a cue is likely to depend not only on context (such as temperature) but also on the shape of the CO_2_ stimulus. Very rapid change in CO_2_ levels may convey a different meaning from a very gradual change. In our behavioral experiments, animals navigated shallow CO_2_ gradients and encountered changes in the order of 0.01% CO_2_ per second (depending on speed and direction of travel in the gradient). To examine if AFD could respond to such shallow CO_2_ gradients, we exposed animals cultivated at 22°C to gradual linear increases and decreases in CO_2_ concentration at rates of 0.04% and 0.01% per second (Figure 3A,B). AFD responded to both these CO_2_ gradients, but with very different response patterns. Gradients of 0.04% CO_2_/second evoked AFD Ca^2+^ responses reminiscent of those elicited by sharp changes in CO_2_ (>1% CO_2_/second; see Figure 2): Ca^2+^ levels decreased while CO_2_ was slowly rising to 5%, then rose sharply as CO_2_ levels stabilized at 5%. When we gradually reduced CO_2_ levels back to 0%, Ca^2+^ levels spiked, returning to baseline when animals were in 0% CO_2_ (Figure 3A). By contrast, gradients of 0.01% CO_2_/second evoked a series of Ca^2+^ spikes while CO_2_ levels were rising (Figure 3B). Ca^2+^ levels tended to return to baseline when CO_2_ levels stopped rising, but spiking resumed when CO_2_ levels started falling. This spiking pattern disappeared when we imaged Ca^2+^ responses evoked by the same 0.01% CO_2_/second gradient in animals acclimated to 15°C (Figure 3C). In these animals responses were more similar to those evoked by steeper CO_2_ gradients in animals acclimated to 15°C (compare Figure 3C to Figure 2B). These data indicate that AFD neurons respond to both rapid and slow changes in CO_2_, but with different response patterns. The also highlight complexity in how AFD encodes CO_2_ stimuli.

### Ambient O_2_ levels regulate *C. elegans* CO_2_ avoidance behaviour

To investigate further how different homeostatic responses are integrated, we examined if CO_2_ avoidance behavior was modulated by different background ambient [O_2_]. In body fluids and many ecological niches low [O_2_] coincides with high [CO_2_], and, conversely, 21% O_2_ is associated with low CO_2_. Cross-talk between the two gas sensing circuits could enable *C. elegans* to recognize and respond appropriately to such environments.

**Figure 4:**
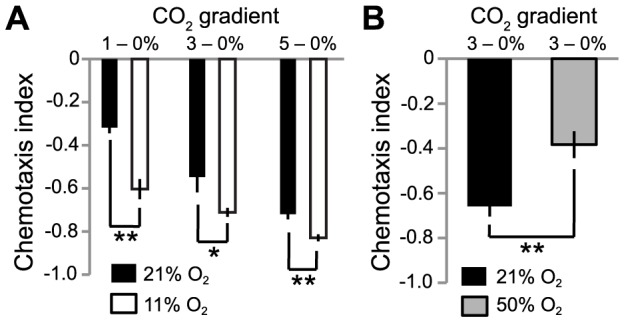
Ambient O_2_ levels set CO_2_ avoidance. A. *C. elegans* avoids shallow gradients of CO_2_ more strongly when O_2_ levels are low. The CO_2_ gradients used are indicated above the graph. B. Artificially high O_2_ levels can reduce CO_2_ avoidance further. **, *p*<0.01; *, p<0.05, Student's *t*-test.

To examine this possibility, we placed N2 animals in microfluidic chambers containing gradients of CO_2_ at different fixed concentrations of O_2_. As expected, increasing [CO_2_] elicited increasing avoidance behavior: *C. elegans* avoided 5% CO_2_ more strongly than 3% or 1% CO_2_ (Figure 4A) [25], [26]. Moreover, CO_2_ avoidance was influenced by the background ambient O_2_ concentration. N2 animals navigated down CO_2_ gradients more strongly when ambient O_2_ concentration was 11%, than when it was 21%. Increased avoidance was particularly striking when animals navigated shallow gradients of 1–0% CO_2_ (Figure 4A). Such shallow CO_2_ gradients are likely to be ecologically relevant in the rotting habitats where *C. elegans* thrives.

To test the dynamic range of O_2_ regulation, we asked if increasing [O_2_] to above 21% could further suppress CO_2_ avoidance. Although this is unphysiological, previous studies have shown that *C. elegans* can grow and reproduce in even 100% O_2_ without any apparent adverse effects [35]. Since *C. elegans* only weakly avoided 1% CO_2_ in 21% O_2_, we used a steeper 3–0% CO_2_ gradient, to improve our dynamic range. Increasing ambient [O_2_] to 50% significantly suppressed avoidance of 3% CO_2_ (Figure 4B). These data suggest that ambient O_2_ concentration provides a contextual cue to modulate *C. elegans* avoidance of CO_2_.

### Tonically signalling O_2_ sensors inhibit CO_2_ avoidance at high ambient [O_2_]

**Figure 5:**
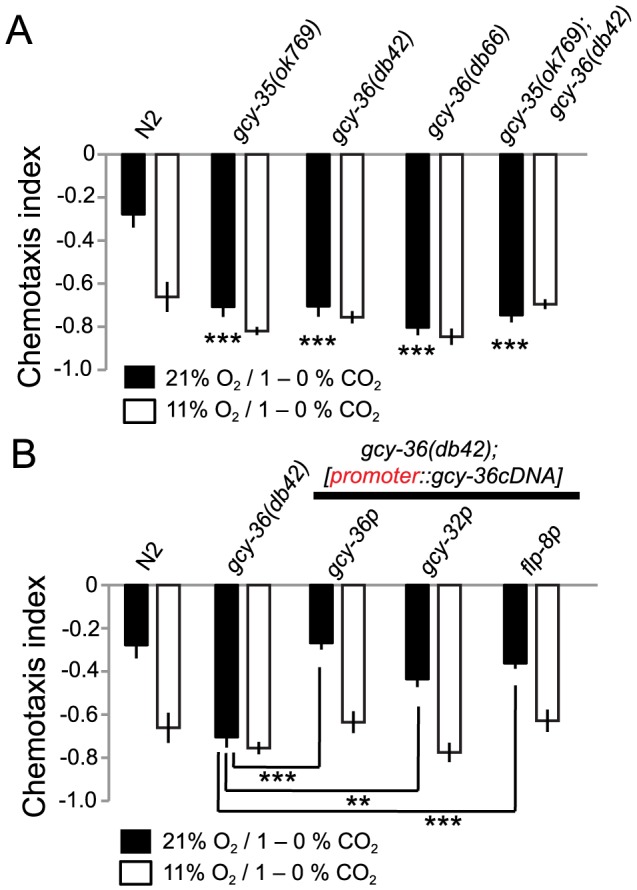
Disrupting *gcy-35* or *gcy-36* confers strong CO_2_ avoidance regardless of ambient O_2_. A. *gcy-35* or *gcy-36* mutants strongly avoid the high CO_2_ half of a 1–0% CO_2_ gradient regardless of ambient O_2_. Statistics refer to comparisons to N2 at 21% O_2_. ***, *p*<0.001, Anova, Bonferroni corrected *p*-value. None of the strains apart from N2 show significant differences between assays carried out at 21% and 11% O_2_ (Student's *t*-test). B. The CO_2_-avoidance phenotype of *gcy-36* mutants can be rescued by expressing *gcy-36* cDNA in AQR, PQR and URX, using *gcy-32* or *gcy-36* promoters, or in URX alone, using the *flp-8* promoter. **, *p*<0.01, ***, *p*<0.001, Anova, Bonferroni corrected *p*-value.

Our results suggested that O_2_-sensing neurons or neuroendocrine cells persistently signal O_2_ concentration to modify the activity of CO_2_ transducing circuits. Previous studies have shown that the AQR, PQR and URX O_2_ sensors signal tonically when ambient [O_2_] is close to 21%, and become progressively less active as [O_2_] falls [24]. The O_2_–evoked Ca^2+^ responses of these neurons requires the atypical soluble guanylyl cyclases GCY-35 and GCY-36, which appear to be O_2_ sensors [20], [22], [36]. In *gcy-35* or *gcy-36* loss-of-function mutants the Ca^2+^ levels in the O_2_ sensing neurons reported by cameleon YC3.60, are low, resembling those found in wild type animals kept at low [O_2_] [24]. To test if tonic signalling by AQR, PQR and URX neurons persistently repressed CO_2_ avoidance in high [O_2_], we compared the CO_2_ avoidance of wild type, *gcy-35*, and *gcy-36* mutants at 21% and 11% O_2_. In 11% O_2_ *gcy-35* and *gcy-36* mutants avoided CO_2_ like N2 controls. However, whereas increasing background O_2_ levels to 21% inhibited the CO_2_ avoidance behavior of wild type animals, it had no effect on *gcy-35* or *gcy-36* mutant animals (Figure 5A). These data suggest that tonic signalling from one or more of the AQR, PQR and URX O_2_ sensors represses CO_2_ avoidance at high O_2_ concentrations.

To confirm our results, we rescued the *gcy-36* mutant phenotype using cell-specific promoters. Expressing *gcy-36* cDNA from its own upstream sequence, which drives expression in AQR, PQR and URX, restored to *gcy-36* mutants reduced CO_2_ avoidance at 21% O_2_ (Figure 5B). *gcy-36* mutants expressing *gcy-36* cDNA from the *gcy-32* promoter, which also drives expression in AQR, PQR and URX, gave similar rescue (Figure 5B). Expressing *gcy-36* cDNA from the *flp-8* promoter, which drives expression in URX (and AUA and PVM) neurons but not in AQR and PQR also rescued the O_2_-regulated CO_2_ avoidance phenotype of *gcy-36* mutants. These results suggest that tonic signalling by the URX O_2_-sensing neuron can persistently suppress CO_2_ avoidance while O_2_ levels are high.

To extend our results we also examined the consequence of deleting *gcy-32* and *gcy-34*, atypical soluble guanylate cyclases expressed in AQR, PQR and URX neurons whose activities are also likely to be modulated by O_2_, but whose deletion only subtly alters O_2_-evoked behaviors. We observed no effects of these deletions on O_2_ regulation of CO_2_ avoidance (Figure S2). We did however observe a slight decrease in CO_2_ avoidance at 11% O_2_ in mutants defective in *gcy-33*, an atypical soluble guanylate cyclase required for the BAG sensory neurons to respond to decreases in O_2_ levels (Figure S2) [22]. BAG is also a major CO_2_ sensor [28] [34].

### The *npr-1* and *glb-5* genes modulate CO_2_ avoidance by O_2_

O_2_ responses in the standard laboratory N2 strain differ from those of aggregating wild *C. elegans*, due to genetic differences that have evolved during domestication [19], [20], [23], [36], [37]. N2 animals harbor a gain-of-function allele of the *npr-1* neuropeptide receptor that inhibits signalling output from O_2_-sensing circuits in feeding animals. N2 animals also carry a loss-of-function mutation in the neuroglobin *glb-5* that increases the excitability of the AQR, PQR and URX O_2_ sensors.

**Figure 6:**
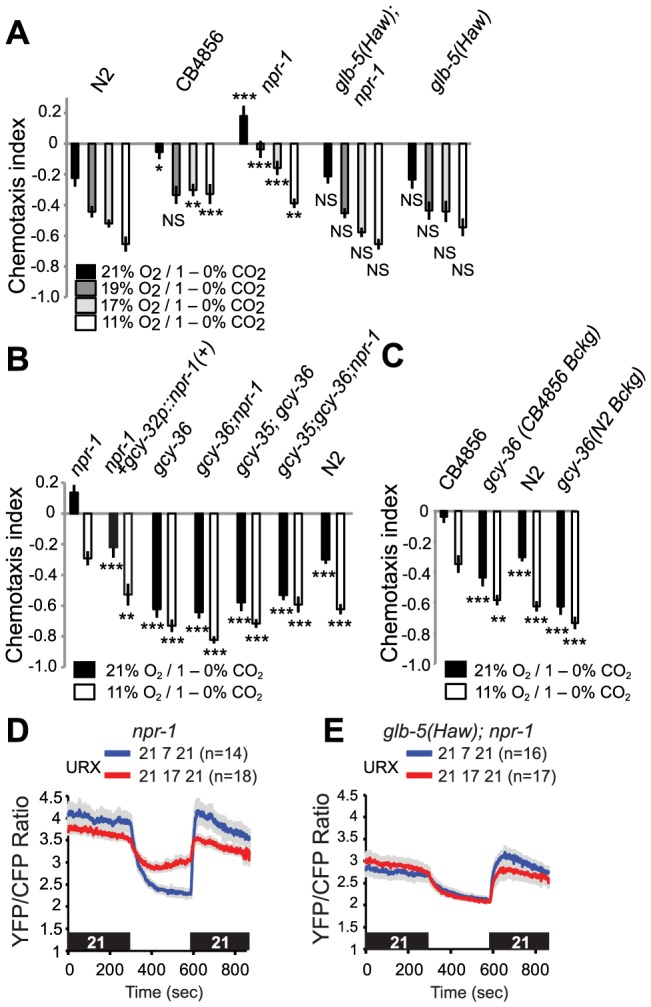
Re-configuring O_2_ sensing circuits by altering the *npr-1* and *glb-5* genes alters CO_2_ avoidance behavior. A. Tuning of CO_2_ avoidance behavior by different O_2_ concentrations in N2 (Bristol), CB4856 (Hawaiian), *npr-1(ad609)*, *glb-5(Haw)*; *npr-1(ad609)*, and *glb-5(Haw)* animals. All assays used a 1–0% CO_2_ gradient. Statistical comparisons are to the N2 response at the corresponding O_2_ concentration, *** *p*<0.001; ** *p*<0.01; *p<0.05 (Anova, *p* value protected Fisher's LSD). B. *gcy-35* and *gcy-36* mutants strongly avoid CO_2_ regardless of genotype at the *npr-1* locus. Statistical comparisons are to the *npr-1* response at the corresponding O_2_ concentration. ***, *p*<0.001, **, *p*<0.01, Anova, Bonferroni corrected *p* value). C. CB4856 (Hawaii) animals defective in *gcy-36* strongly avoid CO_2_ regardless of O_2_ levels. Statistical comparisons are to the CB4856 response at the corresponding O_2_ concentration. ***, *p*<0.001, **, *p*<0.01, Anova, Bonferroni corrected *p* value). D, E. Tonic Ca^2+^ levels in URX neurons of *glb-5(Haw); npr-1* animals kept at 21% O_2_ and 17% O_2_ is lower than Ca^2+^ levels in URX in *npr-1* animals kept at the corresponding O_2_ concentrations. Ca^2+^ measurements were made using cameleon YC2.60.

We investigated if variation at *npr-1* and *glb-5* altered O_2_ modulation of CO_2_ avoidance. In N2 animals, stepwise increases in O_2_ from 11% to 21% caused stepwise decreases in CO_2_ avoidance (Figure 6A). Animals defective in both the *npr-1* receptor and the *glb-5* neuroglobin (i.e. *npr-1* mutants) were attracted to CO_2_ at 21% O_2_, but became progressively more repelled by CO_2_ as O_2_ concentrations fell. A functional *glb-5(Hawaii)* allele made CO_2_ more aversive to *npr-1* defective animals: decreasing [O_2_] still stimulated CO_2_ avoidance, but at each concentration tested *glb-5; npr-1* animals avoided CO_2_ more strongly than *npr-1* animals (Figure 6A). Adding the functional *glb-5(Hawaii)* allele to N2 animals bearing the *npr-1* gain-of-function allele did not significantly change their CO_2_ avoidance behaviour at any O_2_ tensions. Thus, variation at the *glb-5* and *npr-1* genes, which alter O_2_ sensing circuits, changes the extent to which O_2_ levels modifies CO_2_ aversiveness.

To investigate how O_2_ modified CO_2_ avoidance in a non-domesticated *C. elegans* strain, we examined the responses of animals from the Hawaiian CB4856 isolate. As reported previously [23], [25], [26], the Hawaiian strain showed weaker CO_2_ avoidance than N2 at 21% O_2_. Reducing O_2_ levels to 19% was sufficient to strongly stimulate CO_2_ avoidance in Hawaiian animals, and further decreases in [O_2_] had no significant effects (Figure 6A, C). Together, these data suggest that the Hawaiian animals do not avoid CO_2_ when O_2_ is at 21%, i.e. when animals are at the surface, and but that very small decreases in O_2_ are sufficient to increase CO_2_-avoidance behavior. The sharp tuning of CB4856 responses to CO_2_ by O_2_ levels appears to involve the natural alleles of *npr-1*, *npr-1 215F*, the *glb-5(Haw)* alleles.

To shed further light on the genetic control of this cross-talk of CO_2_ and O_2_ responses, we examined how knocking out the soluble guanylate cyclases *gcy-35* and *gcy-36* altered CO_2_ responses in different genetic backgrounds. Knocking out either soluble guanylate cyclase strongly stimulated CO_2_ avoidance in *npr-1* animals: the avoidance behaviour of *gcy-35; npr-1* or *gcy-36; npr-1* animals resembled that of *gcy-35* or *gcy-36* mutants, and of N2 animals at 11% O_2_ (Figure 6B). We also examined the effect of disrupting *gcy-36* in the Hawaiian genetic background (Figure 6C). CB4856 animals defective in *gcy-36* avoided CO_2_ much more strongly than CB4856 controls, and changing ambient O_2_ had little effect on their CO_2_ responses (Figure 6C). Thus, the modulation we describe in domesticated N2 also occurs in wild aggregating *C. elegans*. Expressing cDNA encoding the *npr-1 215V* allele found in N2 animals in the AQR, PQR and URX neurons, using the *gcy-32* promoter, restored N2-like behaviour to *npr-1* mutants (Figure 6B). Thus, *npr-1* acts in the O_2_-sensing neurons themselves to counter the inhibitory effect of high O_2_ on CO_2_ avoidance.

To provide a neural explanation for why *npr-1* animals avoided CO_2_ less than *glb-5(Haw); npr-1* animals at 17%, 19% and 21% O_2_ (Figure 6A, *p*<0.0001, Anova, Bonferroni-corrected *p* value at all three O_2_ values), we compared tonic Ca^2+^ signalling in URX at different O_2_ concentrations. While URX Ca^2+^ levels were similar in *npr-1* and *glb-5; npr-1* animals at 7% O_2_, Ca^2+^ was higher in *npr-1* than in *glb-5; npr-1* animals at 21% and 17% O_2_, consistent with greater inhibition of CO_2_ avoidance by URX signalling at these O_2_ concentrations (Figure 6D, E).

### O_2_ can modulate CO_2_ avoidance in animals defective in AFD and BAG CO_2_ sensors

CO_2_ avoidance in *C. elegans* is mediated by a distributed set of sensory neurons that includes the BAG O_2_ sensor, the AFD temperature sensor, and the ASE gustatory neuron [28], [34]. To examine if O_2_ levels modified CO_2_-evoked Ca^2+^ responses in any of these neurons we imaged their responses at 11% and 21% O_2_ concentrations using the YC3.60 sensor (Figure S3A–C). We did not observe any differences between CO_2_-evoked responses at the two O_2_ concentrations in any of the three neurons under our imaging conditions. This suggests either that O_2_ modulation occurs downstream of these sensory neurons, or that our imaging conditions limit our ability to observe modulation by O_2_.

**Figure 7:**
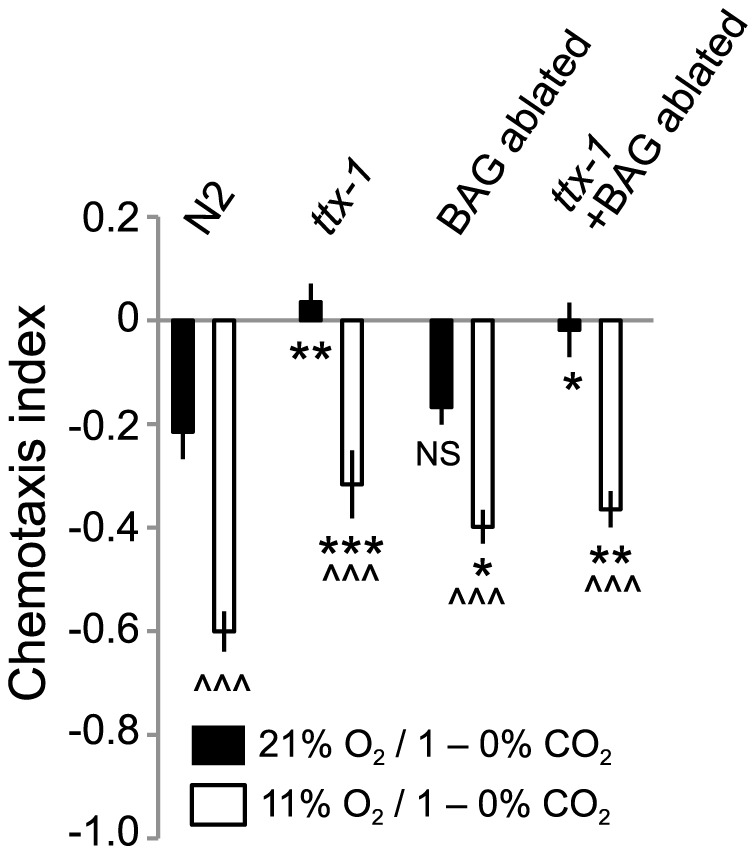
Ambient O_2_ can modulate CO_2_ avoidance in animals lacking BAG and AFD CO_2_ sensors. Animals in which BAG neurons are ablated by specific expression of *egl-1* caspase, and AFD neurons are defective due to loss of *ttx-1*, retain O_2_-modulation of CO_2_ avoidance. *egl-1* expression in BAG neurons is driven by the *flp-17* promoter. ∧∧∧, *p*<0.001, Student's *t* test, comparing a strain's responses at 21% and 11% O_2_. *** *p*<0.001; ** *p*<0.01; * *p*<0.05, Anova, Bonferroni corrected *p* value, comparing responses to that of N2 at the same O_2_ concentration.

O_2_ input could selectively modulate the CO_2_ responses mediated by one CO_2_-sensing neuron, or it could modulate circuits involving multiple CO_2_ sensors. To examine these possibilities, we specifically disrupted AFD and/or BAG function in N2 animals, and measured CO_2_ avoidance at 21% and 11% O_2_. Genetically abating BAG neurons or disrupting AFD specification by mutating the *ttx-1* transcription factor, or doing both, reduced CO_2_ avoidance at 11% O_2_, but did not abolish modulation by ambient O_2_ levels (Figure 7). These data suggest that O_2_ levels either modulate the output from several CO_2_ sensors, or exert their effects on unidentified CO_2_ sensors, or both.

### RIA interneurons are part of the circuit mediating O_2_-modulated CO_2_ avoidance

**Figure 8:**
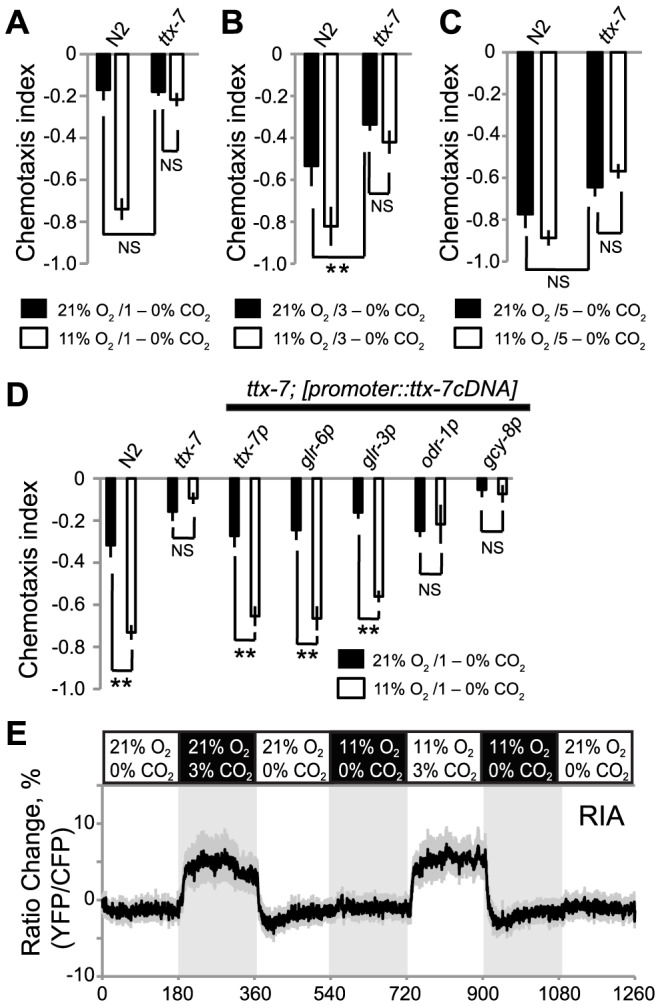
TTX-7 acts in RIA interneurons to promote CO_2_ avoidance when ambient O_2_ levels are low. A–C. Mutations in *ttx-7* strongly reduce CO_2_ avoidance at 11% O_2_ but have relatively weak effects on CO_2_ avoidance at 21% O_2_. ns, not significant, ** *p*<0.01, Student's *t* test. D. Expressing *ttx-7* specifically in RIA neurons, using the *glr-3* or *glr-6* promoters, restores strong CO_2_ avoidance to *ttx-7* mutants assayed at 11% O_2_. Expressing *ttx-7* specifically in AFD, using the *gcy-8* promoter, or in AWB and AWC, using the *odr-1* promoter does not rescue the *ttx-7* phenotype. ns, not significant, ** *p*<0.01, Student's *t* test. E. CO_2_ evokes a Ca^2+^ response in RIA neurons. Ca^2+^ responses were measured in immobilized animals cultivated at 22°C using a *pglr-6::YC3.60* Ca^2+^ reporter. Shading highlights gas switch times. The CO_2_/O_2_ stimulus train used is indicated above the plot.

To dissect further how O_2_-sensing neurons modulated CO_2_ responses, we sought mutations that disrupted O_2_ modulation without abrogating CO_2_ responsiveness. One such mutation we identified was *ttx-7*, which disrupts a *myo*-inositol-1-monophosphatase [38]. *ttx-7* mutants showed only mild defects in CO_2_ avoidance when assayed at 21% O_2_ (Figure 8A–C). The chemotaxis index of *ttx-7* mutants was not significantly different from that of N2 controls when animals were assayed in 1–0% and 5–0% CO_2_ gradients; we only observed a small but significant decrease in CO_2_ avoidance when *ttx-7* mutants were assayed in 3–0% CO_2_ gradients. However, *ttx-7* mutant animals did not increase their CO_2_ avoidance when assayed at 11% O_2_, regardless of the CO_2_ gradient we used (Figure 8A–C). *ttx-7* mutants behaved indistinguishably from N2 animals when assayed in O_2_ gradients (Figure S4), suggesting they were not generally defective in O_2_-evoked responses.

To confirm that the defect in O_2_-dependent modulation of CO_2_ avoidance was due to the *ttx-7* mutation, we showed we could restore strong CO_2_ avoidance at 11% O_2_ to *ttx-7* mutants by expressing *ttx-7* cDNA from the *ttx-7* promoter (Figure 8D). Together, these data suggest that *ttx-7* mutants can sense and respond to O_2_ but cannot communicate information about ambient [O_2_] to the appropriate circuits that mediate CO_2_ responses.

To identify neurons where *ttx-7* acts to promote CO_2_ avoidance at low [O_2_] we rescued the *ttx-7* CO_2_ avoidance phenotype by driving *ttx-7* cDNA in small subsets of neurons. We focussed on neurons that receive synaptic input from the URX O_2_ sensors, since our *gcy-36* rescue experiments implied that URX was sufficient for O_2_ to modulate CO_2_ avoidance (Figure 5B). URX neurons make several synapses onto the RIA interneurons [39]. In turn, RIA neurons receive direct or indirect inputs from many sensory neurons, and are connected to numerous downstream interneurons, making them good candidates for transmitting information about ambient O_2_ to CO_2_ circuits. Previous work has shown that *ttx-7* is required in the RIA neurons to promote appropriate synapse formation and to enable *C. elegans* to navigate temperature gradients [38]. Expressing *ttx-7* cDNA from the *glr-3* or *glr-6* promoters, which drive expression exclusively in RIA [40], restored strong CO_2_ avoidance at 11% O_2_ (Figure 8D). By contrast, *ttx-7* expression in AFD, using the *gcy-8* promoter, or in AWC and AWB olfactory neurons, using the *odr-1* promoter, did not. These data suggest that RIA interneurons are involved in communicating information from O_2_-sensing neurons and/or CO_2_-responsive circuits, to enable its integration.

We examined if CO_2_ elicited a Ca^2+^ response in RIA interneurons, and if this response was modulated by O_2_ context. We exposed animals expressing cameleon YC3.60 in RIA to a stimulus train in which we sequentially altered O_2_ and CO_2_ levels, and measured fluorescence changes in the cell body. 3% CO_2_ evoked a Ca^2+^ response in RIA neurons that was not significantly altered by background O_2_ (Figure 8E). These data suggest that RIA interneurons form part of a CO_2_ responsive circuit. Our inability to detect modulation of CO_2_-evoked Ca^2+^ responses in RIA by O_2_ levels could reflect a limitation of our imaging conditions. Alternatively, O_2_ could regulate RIA independently of Ca^2+^ entry, or could act on neurons downstream of RIA.

### Acclimation temperature and ambient O_2_ act combinatorially to regulate CO_2_ responsiveness

**Figure 9:**
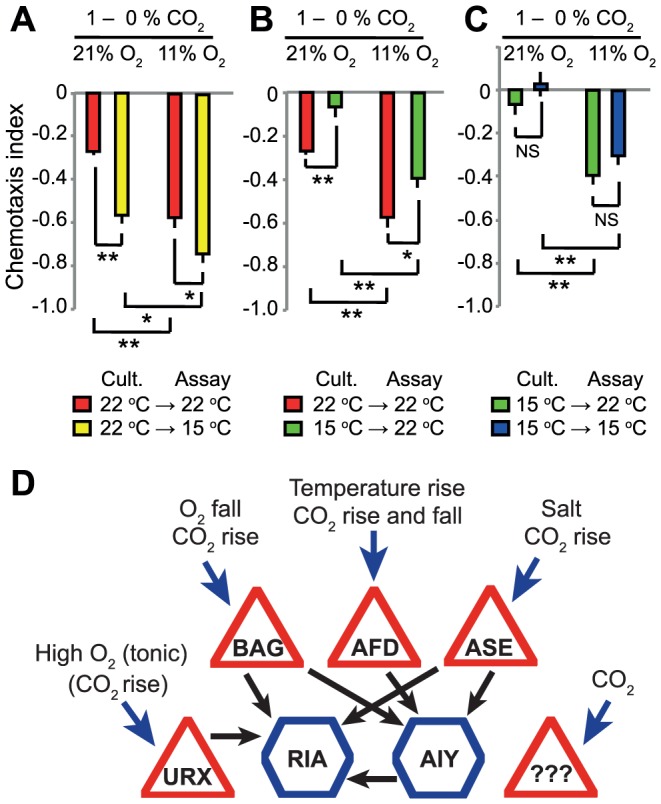
Acclimation temperature and ambient O_2_ levels have additive effects on CO_2_ avoidance. A. Animals cultivated at 22°C but assayed at 15°C avoid CO_2_ more strongly when ambient O_2_ is low. B–C. Reducing O_2_ levels from 21% to 11% increases CO_2_ avoidance regardless of acclimation temperature or assay temperature. In A–C, ** *p*<0.01, * *p*<0.05, ns, not significant, Student's *t* test. D. Coalitions of CO_2_ sensors elicit CO_2_ escape responses according to O_2_ environment, temperature experience, and CO_2_ stimulus dynamics. Triangles represent sensory neurons and hexagons interneurons. Black arrows indicate synapses. Several neurons respond to CO_2_ (blue arrows), each with distinct kinetics. Each of these neurons also responds to other sensory cues, as indicated. Three of the four identified CO_2_ sensors synapse directly onto the RIA interneuron. The fourth, AFD, synapses onto AIY which in turn synapses on RIA. The URX O_2_ sensor also synapses onto RIA. Note each neuron makes additional connections besides the ones highlighted here.

Both acclimation temperature and acute ambient O_2_ concentrations altered *C. elegans'* responsiveness to CO_2_. We investigated how animals integrated information from all three homeostatic systems – temperature, O_2_ and CO_2_. We grew animals at either 15°C or 22°C, and then assayed CO_2_ responses at 15°C or 22°C in the presence of either 21% or 11% O_2_. Our results suggest that the temperature and O_2_ sensing systems act additively to set CO_2_ responsiveness. Decreasing O_2_ from 21% to 11% enhanced avoidance of 1% CO_2_ regardless of acclimation temperature and assay temperature (Figure 9A–C). Similarly, acclimating animals to 15°C decreased avoidance of 1% CO_2_ at both 21% and 11% O_2_ (Figure 9A–C). As described previously (Figure 1A), animals acclimated to 22°C avoided a 1%–0% CO_2_ gradient more strongly when assayed at 15°C rather than 22°C. Changing O_2_ from 21% to 11% further stimulated CO_2_ avoidance in these animals. These data highlight how *C. elegans* homeostatic responses are intertwined with each other.

## Discussion

Previous acclimation temperature and current ambient O_2_ levels set the aversiveness of CO_2_ to *C. elegans*. The temperature animals have experienced previously appears to modify CO_2_ responsiveness by changing the CO_2_ receptive properties of AFD. Acute ambient O_2_ controls CO_2_ preference by regulating tonic signaling from the O_2_ sensing neuron URX. Changes in CO_2_ responsiveness can be observed in shallow gradients with peak CO_2_ levels of 1%. Such gradients are likely to be ecologically relevant for *C. elegans* in the rotting fruit habitats where they are commonly found [41].

*C. elegans* can thrive at temperatures that span ∼15°C–25°C. Within this range, well-fed animals migrate to temperatures at which they were previously growing [13], [29]. Temperature preference appears to be encoded in the AFD neurons: acclimation temperature changes the threshold at which rising temperature evokes Ca^2+^ responses in this neuron [17], [18]. We find that AFD neurons are required for temperature experience to change *C. elegans'* CO_2_ responsiveness. Acclimation temperature qualitatively reconfigures CO_2_-evoked Ca^2+^ responses of AFD neurons. This re-configuration is retained in mutants defective in synaptic release, suggesting it can occur cell-autonomously. A speculative explanation of our observations is that AFD harbors multiple CO_2_ sensors whose contribution to the CO_2_-evoked Ca^2+^ response varies according to acclimation temperature.

AFD neurons are exquisitely sensitive to CO_2_. They respond robustly to changes in CO_2_ that range from <0.01% CO_2_/sec to >1% CO_2_/sec. Remarkably, in animals acclimated to 22°C, the Ca^2+^ responses evoked in AFD by slow (0.01% CO_2_/second) and faster (0.04% CO_2_/second) changes in CO_2_ are qualitatively different. This may explain previous observations that AFD promotes CO_2_ avoidance in shallow CO_2_ gradients, but can inhibit CO_2_ avoidance in steep ones [28].

*C. elegans* avoid CO_2_ less strongly at high O_2_ than at low O_2_. Ambient O_2_ levels provide a contextual cue that modulates the aversiveness of CO_2_. We use the term ‘contextual’ because modulation can occur when O_2_ levels are constant, and is sustained over many minutes. Contextual modulation by O_2_ levels can be graded: as O_2_ decreases from 21% to 11%, CO_2_ avoidance rises. Modulation of CO_2_ avoidance by O_2_ requires the *gcy-35* and *gcy-36* soluble guanylate cyclases, which act in the O_2_ sensing neurons AQR, PQR and URX to transduce O_2_ levels. *gcy-35* or *gcy-36* mutants behave like animals kept at low O_2_, regardless of actual O_2_ levels. The activity of the URX neurons alone appears sufficient to inhibit CO_2_ avoidance at 21% O_2_. Previous work has shown that URX neurons are tonically activated by high O_2_ [24], explaining the ability of these neurons to convey O_2_ context persistently to CO_2_ sensing circuits.

Modulation of CO_2_ avoidance by O_2_ levels can be observed when N2 (Bristol), *npr-1*, *glb-5(Haw); npr-1*, or CB4856 (Haw) animals navigate 1%–0% CO_2_ gradients. However, the degree of inhibition varies across these genotypes. In N2 animals, the inhibitory effect of O_2_ is limited by the action of the NPR-1 215V isoform in O_2_-sensing neurons. *npr-1 215V* does not appear to alter the excitability of O_2_ sensors, since N2 and *npr-1* mutants show similar O_2_-evoked Ca^2+^ responses in URX, AQR or PQR ([22] and data not shown). Instead, we speculate that NPR-1 215V inhibits neurotransmission from URX, for example through G_o_ signaling [42], [43], thus limiting the ability of URX to inhibit CO_2_ responsiveness. Previous work has highlighted coupling of NPR-1 215V to G_o_ in heterologous systems [44]. The potent O_2_-dependent inhibition of CO_2_ avoidance found in *npr-1* mutants is suppressed by the *glb-5(Haw)* allele. This suppression appears to reflect a reduction in the excitability of URX. Tonic Ca^2+^ levels in URX in *glb-5; npr-1* animals kept at 21% O_2_ was only as high as that found in *npr-1* animals at 17% O_2_. In the CB4856 (Haw) strain the combination of the *npr-1 215F* and *glb-5(Haw)* alleles (potentially modified by other loci) enables a switch from 21% to 19% O_2_ to convert CO_2_ from a neutral to a strongly aversive stimulus. While this paper was in preparation independent work also highlighted modulation of CO_2_ avoidance by O_2_ in *npr-1* animals [45]. The assays used are different. Notably, in most of our work we used 1–0% CO_2_ gradients, whereas Carrillo et al. used 10%–0% gradients.

CO_2_ sensing in *C. elegans* is distributed across multiple sensory neurons, including the AFD and BAG neurons [28] (Figure 9D). Disrupting AFD and BAG abolishes CO_2_ avoidance at 21% O_2_, but CO_2_ avoidance at 11% O_2_ is only partly reduced. Thus, CO_2_ sensing neurons other than BAG and AFD can promote CO_2_ avoidance at low O_2_. O_2_ modulation of CO_2_ responsiveness involves the RIA interneurons. *ttx-7* mutants disrupt O_2_ modulation of CO_2_ responsiveness, and expressing *ttx-7* cDNA selectively in RIA neurons rescues this phenotype. *ttx-7* encodes *myo*-inositol monophosphatase. In *ttx-7* mutants RIA neurons exhibit defects in localization of both pre- and post-synaptic components, including synaptobrevin, SYD-2 Liprin, and the glutamate receptor GLR-1 [38]. Synaptic communication via RIA is thus likely to be compromised in *ttx-7* mutants, and may explain the O_2_/CO_2_ integration phenotype.

Previous studies of context-dependent changes in behavior in *C. elegans* have focused mainly on the effects of food or of food deprivation. *C. elegans'* migration in salt and odor gradients can switch from attraction to repulsion if animals are deprived of food in the presence of the chemical cue [46]–[49]. Food and food deprivation have also been shown to modulate *C. elegans* response to temperature gradients [50]. It remains to be seen if acclimation temperature and ambient O_2_ levels have effects on other sensory modalities besides CO_2_ sensing. Whether CO_2_ itself can act as a contextual cue regulating other *C. elegans* sensory responses, including thermotaxis and O_2_ sensing, is also unknown.

The shallow CO_2_ gradients we study are likely to be common in the rotting fruit environments where *C. elegans* is frequently found. However, the ubiquitous production of CO_2_ by aerobically respiring organisms means its value as a sensory cue likely depends crucially on context. Bacterial food, bacterial pathogens, predators, mates and conspecifics may all generate CO_2_ gradients. Context-dependence of CO_2_ responses has been observed previously. *C. elegans* CO_2_ responses are modulated by food, exposure to hypoxia, and starvation [25]. Moreover, not only context, but also the rate of change in CO_2_ concentration (whether it is slow or rapid), appears to modify the contribution of different CO_2_-sensing neurons to *C. elegans* CO_2_ avoidance behaviors [28]. This complexity is mirrored in insects. For example in *Drosophila* airborne CO_2_ is aversive [51], whereas dissolved CO_2_ is attractive [52]. These properties are encoded by separate chemosensory neurons in the antenna (avoidance of gaseous CO_2_) and taste peg neurons (attraction to carbonation). Avoidance of airborne CO_2_ is inhibited by olfactory odors, presumably to enable flies to approach fermenting fruit [53]. Together, these data suggest CO_2_ sensing is remarkably sophisticated in both worms and flies. CO_2_ has been implicated in ageing in *Drosophila* [54], whereas O_2_-sensing neurons modulate longevity in *Caenorhabditis* [55], consistent with neurons sensing these gases also modulating physiology.

## Materials and Methods

### Strains

Strains were maintained at 22°C with plentiful food using standard methods [56]. Strains used in this work are listed in Supplementary methods.

### Behavioral assays and analysis

Spatial carbon dioxide gradient assays were performed as described, with slight modifications [25], [28]. Briefly, rectangular PDMS chambers with a 33×15×0.2 mm space connected to gas syringes were placed over 100–200 worms on a 9 cm NGM agar plate. Assays ran for 20 minutes and the distribution of worms recorded by counting the number of animals in each of nine equal area divisions as well as in the two spaces at either end of the chamber. Animals were washed three times in a watch glass then transferred to the agar. A chemotaxis index was calculated by subtracting the number of animals in the low carbon dioxide half of the chamber from the number in the high carbon dioxide half and dividing by the total number of animals e.g. (A−B)/(A+B), as shown in Figure 1A. In chemotaxis assays, each data point represents the average of at least eight independent assays performed over three experimental days. Certified gases with indicated concentrations of O_2_ and CO_2_ were obtained from BOC UK Ltd. Assays marked 22°C were carried out at room temperature in a room in which temperature varied 22+/−1°C. Assays marked 15°C were carried out in a small thermostat-controlled room set to 15°C.

Statistical comparisons were carried out using the Student's *t* test or ANOVA, as indicated.

### Molecular biology and germline transformation

Standard methods for molecular biology were used [57]. Cosmid and cDNA subcloning were performed using the Invitrogen Multisite Gateway Three-Fragment Vector Construction Kit.

Germline transformation was by microinjection [58] using 2–20 ng/µl for the DNA to be tested, along with 50 ng/µl pJMZ-lin-15 (+) construct and carrier DNA, pBluescriptII SK (+).

### Ca^2+^ imaging

Ca^2+^ imaging was carried out as described previously [24], [28], using an inverted microscope (Axiovert, Zeiss), a 40× C-Apochromat lens, and MetaVue acquisition software (Molecular Devices).

## Supporting Information

Figure S1 CO_2_-evoked responses in AFD do not require the GCY-9 transmembrane guanylate cyclase (A, B), whereas BAG responses do (C, D). For all experiments animals were grown at 22°C.
(EPS)

Figure S2 Disrupting *gcy-33* reduces CO_2_ avoidance at 11% O_2_, whereas disrupting *gcy-32* or *gcy-34* has no effect on CO_2_ avoidance either at low or high O_2_. *, *p*<0.05, **, *p*<0.01, ns, not significant, Student's *t*-test.
(EPS)

Figure S3 CO_2_-evoked Ca^2+^ responses in ASE (A), BAG (B) and AFD (C) neurons are not altered by background O_2_ levels under our imaging conditions. CO_2_ and O_2_ stimuli are indicated above each plot.
(EPS)

Figure S4 *ttx-7* mutants behave like N2 animals in 21%–0% O_2_ gradients.
(EPS)

Text S1 Strain list.
(DOCX)
