# Supplementary material for: Cross-Modulation of Homeostatic Responses to Temperature, Oxygen and Carbon Dioxide in C. elegans
Source: PLoS Genet. 2013 Dec 19;9(12):e1004011. doi: 10.1371/journal.pgen.1004011 (PMC3868554; doi:10.1371/journal.pgen.1004011)
Supplement: Text S1 — Strain list. (DOCX) [file pgen.1004011.s005.docx]

**Cross-modulation of homeostatic responses in *C. elegans***

Eiji Kodama-Namba^1^, Lorenz A. Fenk^1^, Andrew J. Bretscher^1^, Einav Gross^1^, K. Emanuel Busch^1^, and Mario de Bono^1^

^1^MRC Laboratory of Molecular Biology, Francis Crick Avenue, Cambridge, CB2 0QH, UK.

**Supplementary Information**

**Strain list:**

The wild strains used were N2 (Bristol) and CB4856 (Hawaii). Mutant strains used were in the N2 background unless otherwise indicated. Strains used include: AX1295, *gcy-35(ok769) I*; AX1296, *gcy-36(db42) X*; AX1798, *gcy-36(db42) X* (CB4856 background); AX2396, *gcy-35(ok769) I; gcy-36(db42) X*; RB564, *gcy-31(ok296) X*; RB1048, *gcy-32(ok995) V*; CZ3715, *gcy-33(ok232) V*; RB1062, *gcy-34(ok1012) V*; AX204, *npr-1(ad609) X*; PR767, *ttx-1(p767) V*; AX195, *npr-1(ad609) gcy-36(db42) X*; AX1280, *gcy-35(ok769) I; npr-1(ad609) gcy-36(db42) X*; AX2054, *gcy-31(ok296) gcy-33(ok232) V*; AX1797, *glb-5 V*; AX1891, *glb-5(Haw)* V*; npr-1(ad609) X*; IK589, *ttx-7(nj50) I*; Transgenic strains: AX2399, *gcy-36(db42) lin-15(n765ts) X; Ex [gcy-36 promoter::gcy-36 cDNA::SL2gfp*, *lin-15*(+)*]*; AX2400, *gcy-36(db42) lin-15(n765ts) X; Ex [gcy-32 promoter::gcy-36 cDNA::SL2gfp, lin-15*(+)*]*; AX2401, *gcy-36(db42) ) lin-15(n765ts) X; Ex [flp-8 promoter::gcy-36 cDNA::SL2gfp, lin-15*(+)*]*; PY2137, *ttx-1(p767) V; Ex [14 Kb ttx-1(+), unc-122 promoter::gfp]*; AX2051, *dbEx [gcy-33 promoter::egl-1, unc-122 promoter::dsRed]*; AX2172, *ttx-1(p767) V; dbEx [gcy-33 promoter::egl-1, unc-122 promoter::dsRed]*; AX1907, *lin-15(n765ts) X;* *dbEx* [*gcy-32 promoter::YC3.60, lin-15*(*+*)]; XL76, *lin-15(n765ts) X; ntIs13 [flp-6 promoter::YC2.12, lin-15*(*+*)*]*; AX2073, *lin-15(n765ts) X; dbEx [flp-17 promoter::YC3.60, lin-15*(+)*]*; AX2047, *dbEx [gcy-8 promoter::YC3.60, odr-1 promoter::mCherry]*; YTA571, *ttx-7(nj50) I; Ex [ttx-7 promoter::ttx-7 cDNA, ges-1 promoter::gfp]*; YTA606, *ttx-7(nj50) I; Ex [glr-6 promoter::ttx-7 cDNA, ges-1promoter::gfp]*; YTA562, *ttx-7(nj50) I; Ex [glr-3 promoter::ttx-7 cDNA, ges-1 promoter::gfp]*; YTA577, *ttx-7(nj50) I; Ex [odr-1 promoter::ttx-7 cDNA, ges-1 promoter::gfp]*; YTA574, *ttx-7(nj50) I; Ex [gcy-8 promoter::ttx-7 cDNA, ges-1promoter::gfp]*; AX2405, *dbEx [glr-6 promoter::YC3.60]*.
